# A Better Antiviral Efficacy Found in Nucleos(t)ide Analog (NA) Combinations with Interferon Therapy than NA Monotherapy for HBeAg Positive Chronic Hepatitis B: A Meta-Analysis

**DOI:** 10.3390/ijerph120810039

**Published:** 2015-08-21

**Authors:** Wei Wei, Qinmei Wu, Jialing Zhou, Yuanyuan Kong, Hong You

**Affiliations:** 1 Clinical Epidemiology and Evidence-based Medicine Center, Beijing Friendship Hospital, Capital Medical University, 95 Yong-An Road, Beijing 100050, China; E-Mail: vivi_0306@126.com; 2 National Clinical Research Center for Digestive Diseases, 95 Yong-An Road, Beijing 100050, China; E-Mails: qinmeiw@163.com (Q.W.); zhoujialing11@126.com (J.Z.); 3 Beijing Key Laboratory of Translational Medicine in Liver Cirrhosis, Liver Research Center, Beijing Friendship Hospital, Capital Medical University, National Clinical Research Center for Digestive Diseases, 95 Yong-An Road, Beijing 100050, China

**Keywords:** interferon, nucleos(t)ide analogues, combination therapy, antiviral, chronic hepatitis B

## Abstract

*Background*: The clinical efficacy of nucleos(t)ide analogues (NAs) combined with interferon (IFN) therapy *vs.* NAs monotherapy for chronic hepatitis B (CHB) remains inconclusive. The aim of this meta-analysis was to determine whether the NAs plus IFN regimen offers synergistic efficacy that justifies the cost and burden of such a combination therapy in CHB patients. *Methods*: Related publications covering the period of 1966 to July 2014 were identified through searching MEDLINE, EMBASE, Cochrane library, Chinese Biomedical Literature Database, WANFANG, and CNKI database. A total of 17 studies were enrolled, including 6 in English and 11 in Chinese. Then, we established a final list of studies for the meta-analysis by systematically grading the quality and eligibility of the identified individual studies. We used hepatitis B antigen (HBeAg) loss, HBV-DNA undetectable rate, HBeAg seroconversion, hepatitis B surface antigen (HBsAg) loss, HBsAg seroconversion, and histological score at the end of treatment for efficacy evaluation. A quantitative meta-analysis (Review Manager, Version 5.1.0) was performed to assess the differences between NAs and IFN combination therapy and NAs monotherapy. *Results*: Our analysis demonstrated that HBeAg loss (RR = 1.73, 95% CI = 1.32–2.26, *p* < 0.001), HBV-DNA undetectable rate (RR = 1.58, 95% CI = 1.22–2.04, *p* < 0.001), HBeAg seroconversion (RR = 1.68, 95% CI = 1.36–2.07, *p* < 0.001), and HBsAg loss (RR = 2.51, 95% CI = 1.32–4.75, *p* < 0.001) in the combination therapy group were significantly higher than those in the monotherapy group. However, there were no significant differences in HBsAg seroconversion (RR = 4.25, 95% CI = 0.62–29.13, *p* = 0.14), sustained virological response rates, and biochemical response rates observed between the two groups. The results showed that the combination therapy group had more improved HBV histology than the NAs monotherapy group (RR = 1.14, 95% CI = 0.93–1.39, *p* = 0.22). *Conclusions*: NAs and IFN or Peg-IFN combination therapy had a better efficacy in terms of HBeAg loss, HBV-DNA undetectable rate, HBeAg seroconversion, and HBsAg loss, compared to the NA monotherapy group at the end of treatment; however, there was no significant difference in HBsAg seroconversion between the two regimens.

## 1. Introduction

Approximately 350–400 million people are chronically infected with hepatitis B virus (HBV) worldwide [1]. Chronic HBV infection remains a significant challenge to public health, despite the success of HBV vaccination. Clinical manifestations of chronic HBV infection vary from asymptomatic (hepatitis B antigen (HBeAg) positive) or an inactive carrier (HBeAg negative) state to progressive chronic hepatitis B (CHB) that is either HBeAg positive or negative. Ongoing liver injury in chronic HBV infection can lead to fibrosis, cirrhosis, and hepatocellular carcinoma [2]. Current antiviral therapy is designed to stop the progression of chronic liver injury via inhibiting HBV replication. Over the past several decades, several antiviral drugs have been approved for CHB treatment, including interferon (both conventional and pegylated, IFN) and five nucleos(t)ide analogs (NAs): lamivudine (Lam), adefovir (Adv), telbivudine (LDT), entecavir (ETV), and tenofovir (TDF) [3]. The advantages of IFN therapy consist of shorter treatment duration, the absence of drug resistance, and relatively higher rates of hepatitis B antigen (HBeAg) and hepatitis B surface antigen (HBsAg) seroconversion. The inadequacies of using IFN or PEG-IFN include a moderate antiviral effect, for instance, the sustained virological response was just about 40% in HBeAg-negative patients, inferior tolerability, risk of adverse events and subcutaneous injections [4]. Oral NAs, especially new generation NAs, show potent efficacy in inhibiting HBV DNA replication, normalizing alanine transaminase (ALT) levels, and improving histology. However, the use of NAs relies on long-term therapy and induces drug-related mutant infection, which may or may not be associated with drug-resistant mutant infection breakthrough [5]. HBeAg seroconversion after 48 weeks of therapy remains low, despite the improved inhibition of HBV DNA replication. HBsAg seroconversion varies from 1% to 3% [6]. NAs rarely clear chronic HBV infection, and HBV replication bounces back after cessation of treatment [7]. To minimize deficiencies associated with either NAs or IFN monotherapy, the combination of NAs and IFN for treating chronic HBV infection has been explored in recent years [8]. The reasoning for combination therapy lies in the hope that it may produce therapeutic synergy because different antiviral mechanisms are utilized by NAs and IFN. There have been several dozens of published studies comparing the antiviral efficacy between combination therapy and monotherapy. However, the results among individual trials are understandably inconsistent, and even controversial and confusing. Because of the sporadic nature of published reports spanning almost a decade, it is beneficial to extract and summarize key findings to address critical questions in the field, including: (1) Whether the NAs combination therapy with IFN is more efficacious than NAs monotherapy; and (2) Does the benefit justify the cost and burden incurred to patients by the combination therapy? In this study, we performed a systematic review and meta-analysis of eligible clinical studies to address these two questions.

## 2. Materials and Methods

### 2.1. Literature Search

The systemic literature search was carried out by extracting related publications from the following electronic databases: MEDLINE, EMBASE, Cochrane library, Chinese Biomedical Literature Database, WANFANG, and CNKI, covering the period between 1966 to July 2014. The search strategy was as follows: “((((peginterferon OR peg interferon OR pegylated interferon OR peg-IFN OR peg IFN OR pegasys OR pegintron)) AND (nucleotide analogue $1 OR nucleotide analog $1 OR nucleoside analog $1 OR nucleoside analogue $1 OR NUC OR lamivudine OR Lam OR adefovir OR Adv OR entecavir OR ETV OR tenofovir OR TDF)) AND ((chronic hepatitis b OR CHB OR HBV OR hepatitis B virus OR hepatitis B)))”.

### 2.2. Inclusion and Exclusion Criteria

The following inclusion criteria were applied: (1) HBeAg positive; (2) the trial design was aimed to compare NAs combined with IFN *vs.* NAs monotherapy; and (3) all patients were treatment naive at enrollment and showed good compliance to the prescribed dosage and treatment course. The exclusion criteria were as follows: (1) patients who were co-infected with hepatitis A, C, or D virus, or HIV virus; (2) patients who had decompensated liver disease or hepatocellular carcinoma; (3) patients who had a prior liver transplantation; (4) patients who had comorbidities such as alcoholism, autoimmune disease, or metabolic liver disease; (5) patients who were pregnant.

### 2.3. Study Design

Each of the eligible studies must have been designed to compare the efficacy between the combination therapy and NAs monotherapy. Separate meta-analyses were conducted for each group and the whole patient population. The methodological quality in each of the included studies was assessed with the Jadad scale standard, an established composite score that evaluates randomization, concealment, and reporting of patient withdrawal and dropout rates; scores ≥3 signify high quality studies. Study heterogeneity was evaluated for each analysis.

### 2.4. Efficacy Measures

Loss of HBeAg at the end of the treatment (the median treatment duration was 48 weeks) was used as the primary endpoint in all eligible studies. HBeAg/HBsAg loss means undetectable HBeAg/HBsAg in the serum, and seroconversion refers to the appearance of detectable HBeAg/HBsAg antibodies. The detailed methods for detecting HBeAg are listed in Table 1. The serum HBV-DNA undetectable rate, HBeAg seroconversion, HBsAg loss, HBsAg seroconversion, and histology improvement were used as secondary endpoints. Serum HBV-DNA was quantitatively determined by real-time polymerase chain reaction, which detected viral DNA between 10^2^ and 10^9^ copies/mL. The undetectable level was defined as less than 10^2^ copies/mL. Histology improvement was defined as the reduction of the histological fibrosis score or Ishark score at the end point of treatment, compared to the scores at baseline.

Three NAs, including Lam, ETV, and Adv, were used in combination and as a monotherapy among eligible studies. Thus, three comparisons between the combination therapy and monotherapy were individually made.

### 2.5. Data Extraction

Two authors independently screened the abstracts and extracted the efficacy data using a data extraction form. A third investigator was invited to resolve conflicts. When duplicate or triplicate studies describing similar research were identified, only the most recent and complete study was selected for the data extraction.

### 2.6. Statistical analysis

Quantitative meta-analyses were performed to evaluate differences in the antiviral efficacy between the combination therapy and monotherapy. The collected data were processed by the statistical software Review Manager, version 5.1.0. The risk ratio (RR) was calculated with each respective 95% confidence interval (CI), and these values were presented for each individual study. Heterogeneity was evaluated for each meta-analysis by means of Q-statistics and their corresponding *p* values.

**Table 1:** Characteristics of studies included for this systematic review.

Study	Sample Size	Regimen		Detection Methods for HBsAg and HBeAg	Treatment Duration (Weeks)	Follow-up Period (Weeks)	HBV Genotype		Interferon
	Comb/Mono	Comb	Mono				Comb	Mono	Type
Yuan, 2014 [9]	26/28	ETV 0.5 mg/d + Peg-IFNα-2a 180 μg	ETV 0.5 mg/d	AIA1800 Chemiluminescence Analyzer, TOSOH Corporation, Tokyo, Japan	48	52	unknown	unknown	Peg-IFNα-2a
Zeng, 2013 [10]	20/20	ETV 0.5 mg/d + Peg-IFNα-2a 180 μg	ETV 0.5 mg/d	i2000 electro-chemiluminescence, Abbot Diagnostic Division, Sligo, UK	24	unknown	unknown	unknown	Peg-IFNα-2a
Yu, 2013 [11]	90/45	Lam 100 mg + IFN-α-2b 50 μg/Adv 10 mg + IFN-α-2b 50 μg/Adv 10 mg + Peg-IFNα-2a 180 μg	Lam 100 mg/ Adv 10 mg	Enzyme immunoassay, AxSym analyzer, Abbot Diagnostic Division	96	24	unknown	unknown	IFN-α-2b/Peg-IFNα-2a
Li, 2013 [12]	17/22	Adv 10 mg + Peg-IFNα-2a 180 μg	Adv 10 mg	unknown	48	unknown	unknown	unknown	Peg-IFNα-2a
Li, 2012 [13]	44/51	ETV 10 mg/d + IFNα-2b	ETV 10 mg/d	Cobass601 electro-chemiluminescence, Roche, Basel and Kaiseraugst, Switzerland	48	unknown	unknown	unknown	IFNα-2b
Wang, 2012 [14]	30/31	Adv 10 mg + IFNα-2b 50 μg	Adv 10 mg	IMx analyzer, Abbot Diagnostic Division	48	24	unknown	unknown	IFNα-2b
Chen, 2012 [15]	19/35	ETV 0.5 mg/d + Peg-IFNα-2a 180 μg	ETV 0.5 mg/d	Architect HBsAg QT, Abbot Diagnostic Division	72	72	B 12 (63%) C 7 (37%)	unknown	Peg-IFNα-2a
Ding, 2010 [16]	22/23	Adv 10 mg + Peg-IFNα-2a 180 μg	Adv 10 mg	unknown	48	unknown	unknown	unknown	Peg-IFNα-2a
Piratvisuth, 2008 [17]	112/112	Lam 100 mg + Peg-IFNα-2a 180 μg	Lam 100 mg	Microparticle enzyme immunoassay (AXSYM HBe 2.0, Abbott Laboratories, Abbott Park, IL, USA)	48	48	B 30% C 32%	43% 49%	Peg-IFNα-2a
Li, 2006 [18]	31/43	Adv 10 mg + IFNα-2b 50 μg	Adv 10 mg	Shanghai GeneCore Biotechnologies Corporation, Shanghai, China	104	unknown	unknown	unknown	IFNα-2b
Janssen, 2005 [19]	130/136	Lam 100 mg + Peg-IFNα-2b 100 μg/w	Lam 100 mg	EIA (AxSYM, Abbott, Abbott Park, Chicago, IL, USA).	52	24	A 43 (33%) B 11 (9%) C 18 (14%) D 52 (40%)	47 (35%) 12 (9%) 21 (15%) 51 (38%)	Peg-IFNα-2b
Amarapurkar, 2005 [20]	20/5	Lam 100 mg + Peg-IFNα-2b 180 μg	Lam 100 mg	Versant HBV DNA 1.0 assay, Bayer Corp, NewYork, NY, USA	48	24	unknown	unknown	Peg-IFNα-2b
Sarin, 2005 [21]	38/37	Lam 100 mg + IFN-α 5 MU	Lam 100 mg	Enzyme immunoassay	52	24	unknown	unknown	IFN-α
Chan, 2005 [22]	48/47	Lam 100 mg + Peg-IFNα-2b 100 μg	Lam 100 mg	Applied Biosystems, Foster City, CA, USA	60	52	B 15 (31%) C 30 (62%)	16 (34%) 28 (60%)	Peg-IFNα-2b
Lau, 2005 [23]	271/272	Lam 100 mg + Peg-IFNα-2a 180 μg	Lam 100 mg	Central laboratory, AxSYM test (Abbott)	48	24	A 18 (7%) B 82 (30%) C 156 (58%) D 11 (4%)	15 (6%) 73 (27%) 162 (60%) 17 (6%)	Peg-IFNα-2a
Song, 2004 [24]	60/30	Lam 100 mg + IFNα-2b 3 MU	Lam 100 mg	71705 Biochemical analyzer, HITACHI, Tokyo, Japan	24	60	unknown	unknown	IFNα-2b
Deng, 2003 [25]	32/24	Lam 100 mg + IFNα-2b 5 MU	Lam 100 mg	ELISA, Shanghai Kehua Bio-engineering Corporation, Shanghai, China	48	24	unknown	unknown	IFNα-2b

## 3. Results

### 3.1. Included Studies

**Figure 1:**
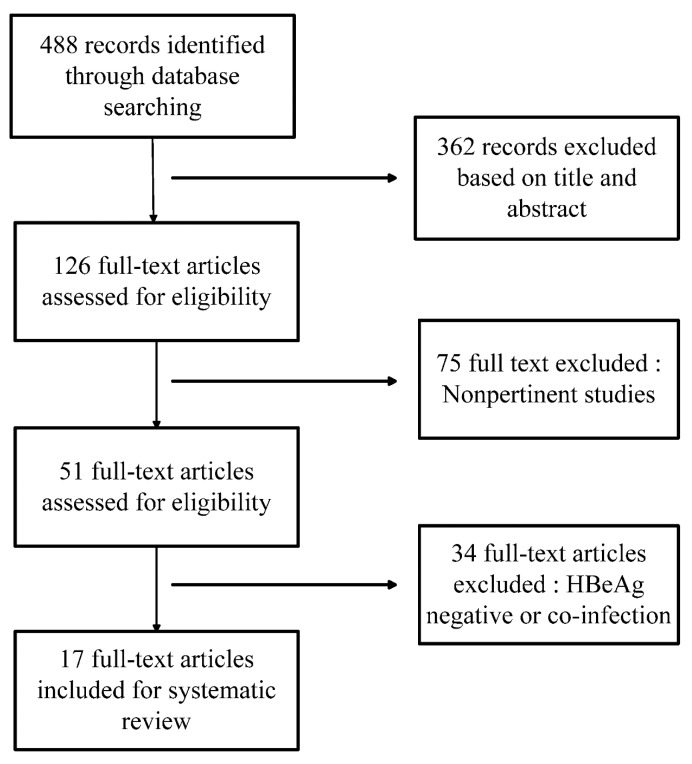
Flowchart of the published work search and selection process.

A total of 488 literature studies were retrieved from all searched electronic databases, and 362 of them were excluded based on analysis of the title and abstract. In addition, 75 nonpertinent studies and 34 studies with coviral infection were excluded after further screening (Figure 1). The remaining 17 studies consisted of 2479 patients in total, of whom 1259 patients received combination therapy and 1220 underwent monotherapy. Of these 17 studies, 6 were published in English and 11 in Chinese. A total of 16 studies performed combination therapy at the beginning of the treatment. Only one study mentioned sequential therapy. All of the patients showed comparable baseline characteristic data between the two groups.

### 3.2. HBeAg Loss

**Figure 2:**
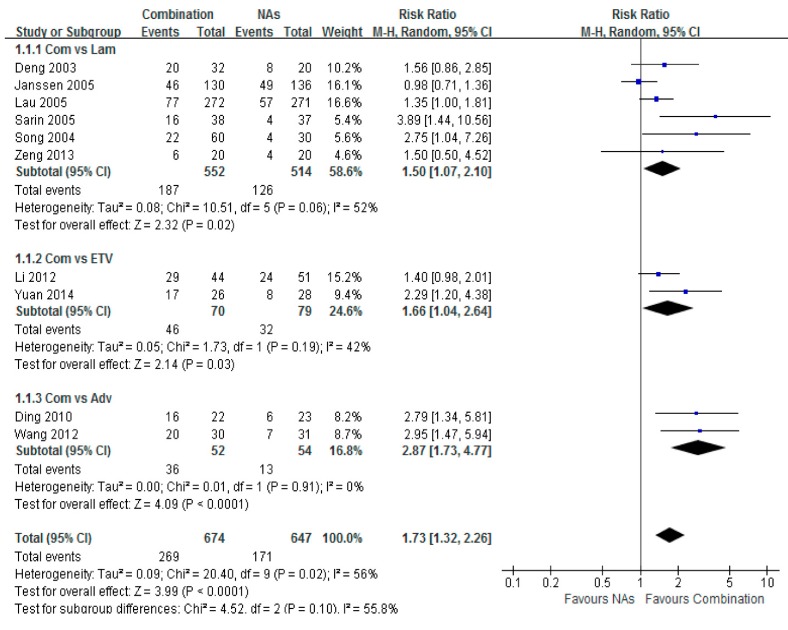
Forest plot of the serum HBeAg loss of combination therapy group *vs.* nucleoside analogues monotherapy group. Eleven trials reported the serum HBeAg loss rate of chronic hepatitis B patients. A higher potent of HBeAg loss was shown in combination group than in monotherapy group. The highest was combination group *vs.* Adv group (RR = 2.87, 95% CI = 1.73–4.77, *p* < 0.001). Total risk ratio was 1.73 (95% CI = 1.32–2.26, *p* < 0.001).

Ten trials reported the serum HBeAg loss rates among treated HBeAg-positive patients. Significant differences were found between the combination therapy and monotherapy groups, with a total risk ratio of 1.73 (95% CI = 1.32–2.26, *p* < 0.001). The highest HBeAg loss was noted between the Adv combination and monotherapy groups, with a risk ratio of 2.87 (95% CI = 1.73–4.77, *p* < 0.001) (Figure 2).

### 3.3. HBV-DNA Undetectable Rate

**Figure 3:**
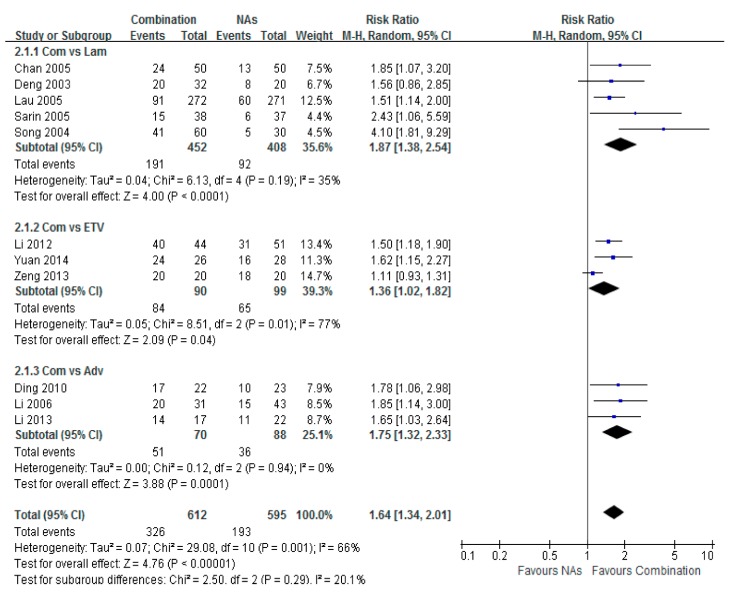
Forest plot of the serum HBV-DNA undetectable rate of combination therapy group *vs.* nucleoside analogues monotherapy (Lam/ETV/Adv) group. The highest serum HBV-DNA undetectable rate was obtained in the combination therapy group compared with Lam. The risk ratio was 1.87 (95% CI = 1.38–2.54, *p* < 0.001). The higher serum HBV-DNA undetectable rate was obtained in the combination therapy group compared with Adv. The risk ratio was 1.75 (95% CI = 1.32–2.33, *p* < 0.001). A significant difference was found between patients in the combination group and ETV group. The risk ratio was 1.64 (95% CI = 1.34–2.01, *p* < 0.001).

Serum HBV-DNA undetectable rates were reported among 13 studies, which provided both the number and the percentage of patients with undetectable HBV DNA. The virological response was analyzed individually among three subgroups based on the NAs modality. The highest serum HBV-DNA undetectable rate was found in the Lam combination therapy group compared with IFN, with a risk ratio of 1.87 (95% CI = 1.38–2.54, *p* < 0.001). The highest serum HBV-DNA undetectable rate was detected in the Adv combination therapy group, with a risk ratio of 1.75 (95% CI = 1.32–2.33, *p* < 0.001). A significant difference was found between the ETV combination group and the monotherapy group, with a risk ratio of 1.64 (95% CI = 1.34–2.01, *p* < 0.001) (Figure 3).

### 3.4. HBeAg Seroconversion

**Figure 4:**
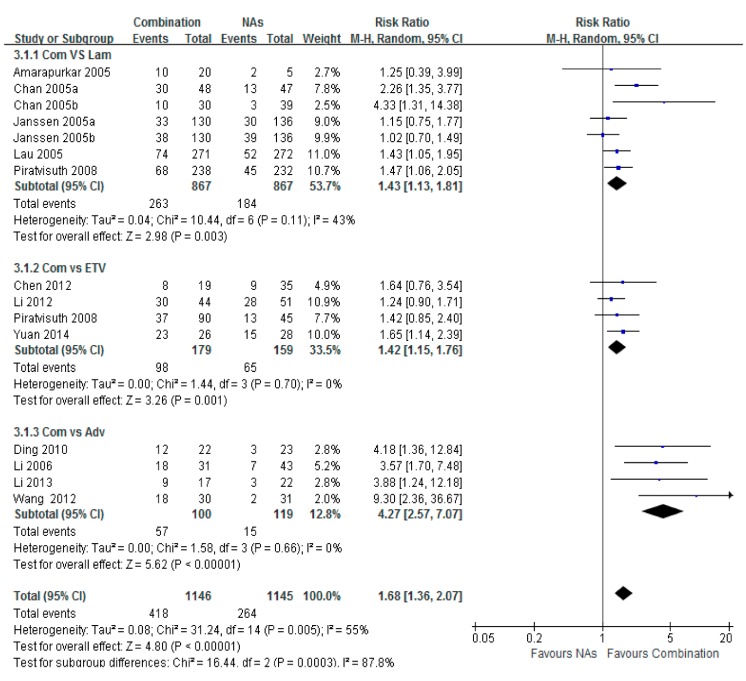
Forest plot of the serum HBeAg seroconversion of combination therapy group *vs.* Lam, ETV and Adv group. Significant difference were observed in combination group between those groups. The highest group was combination *vs.* Adv group, and risk ratio was 4.27 (95% CI = 2.57–7.07, *p* < 0.001). The higher risk ratio of combination *vs.* Lam group was 1.43 (95% CI = 1.13–1.81, *p* = 0.002). The total risk ratio was 1.68 (95% CI = 1.36–2.07, *p* < 0.001).

Significant differences in HBeAg seroconversion were observed between each of the three sets of Lam, ETV, or Adv combination and NA monotherapy. The highest subtotal risk ratio in the Adv combination therapy group *vs.* the Adv monotherapy group was 4.27 (95% CI = 2.57–7.07, *p* < 0.001). The subtotal risk ratio in the ETV combination therapy group *vs.* the Lam group was 1.43 (95% CI = 1.13–1.81, *p* = 0.001). The subtotal risk ratio in the ETV combination therapy *vs.* the Lam group was 1.42 (95% CI = 1.15–1.76, *p* < 0.001), and the total risk ratio was 1.68 (95% CI = 1.36–2.07, *p* < 0.001) (Figure 4).

### 3.5. HBsAg Loss

**Figure 5:**
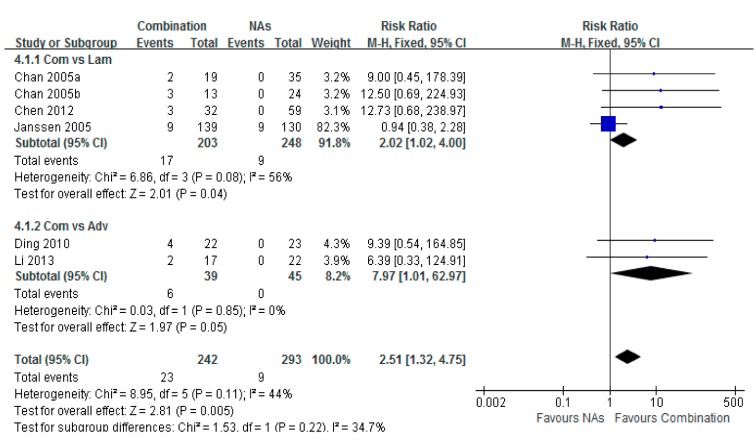
Forest plot of the serum HBsAg loss of combination therapy group *vs.* nucleoside analogues monotherapy group. Seven studies reported the serum HBsAg loss rate of chronic hepatitis B patients. A higher potent of HBsAg loss was shown in combination group than in monotherapy group. Total risk ratio was 2.51 (95% CI = 1.32–4.75, *p* < 0.001).

Six studies reported the serum HBsAg loss rates among treated patients. A higher frequency of HBsAg loss was detected in the pooled patients with combination therapy, compared to those in the monotherapy group. The total risk ratio was 2.51 (95% CI = 1.32–4.75, *p* < 0.001) (Figure 5).

### 3.6. HBsAg Seroconversion

**Figure 6:**
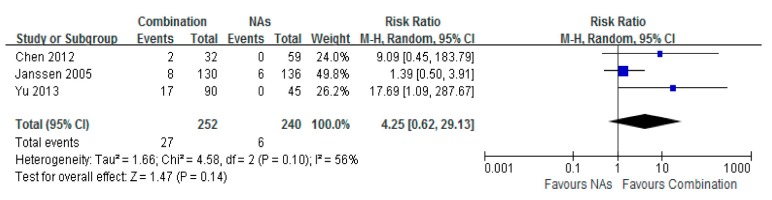
Forest plot of HBsAg Seroconvertlon of combination therapy group *vs.* nucleoside analogues monotherapy group. No significant differences of HBsAg seroconvertion were observed for patients given combination therapy *vs.* monotherapy group respectively. Total risk ratio was 4.25 (95% CI = 0.62–29.13, *p* = 0.14).

No significant difference of HBsAg seroconversion was observed for patients between the Lam combination therapy and monotherapy groups. The total risk ratio was 4.25 (95% CI = 0.62–29.13, *p* = 0.14) (Figure 6).

### 3.7. Histological Improvement

**Figure 7:**
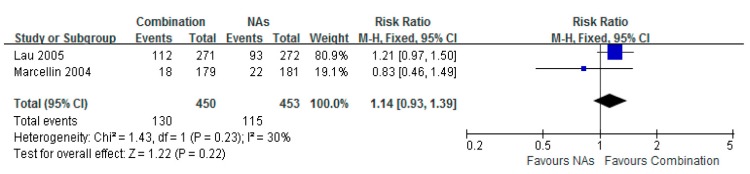
Forest plot of the histologic improvement of combination therapy group *versus* nucleoside analogues monotherapy group. Two studies involved the number of histologic improvement. No significant difference was shown in the two groups. Total risk ratio was 1.14 (95% CI = 0.93–1.39, *p* = 0.22).

Two studies provided data of histology comparison. There was no significant difference in the histological improvement between the Lam combination and Lam monotherapy groups (RR = 1.14, 95% CI = 0.93–1.39, *p* = 0.22). However, the data demonstrated a clearer tendency of improving HBV-induced liver fibrosis in the Lam combination therapy group, compared to Lam therapy alone (Figure 7).

**Figure 8:**
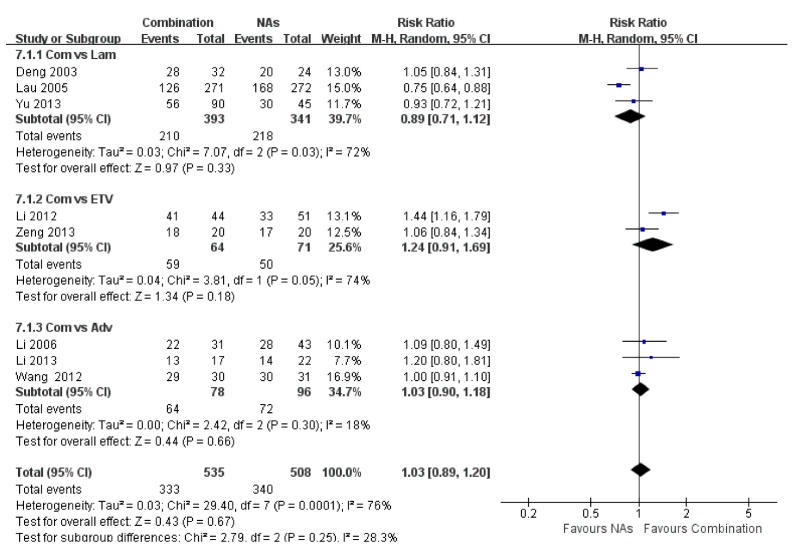
No significant difference in ALT normalization was Identified between the combination and NAs group, and the total risk ratio was 1.03 (95% CI = 0.89–1.20, *p* = 0.67).

### 3.8. ALT Normalization

Seven studies provided data regarding the percentages of ALT normalization at the end of treatment. No significant difference in ALT normalization was identified between the combination therapy and NA monotherapy groups, and the total risk ratio was 1.03 (95% CI = 0.89–1.20, *p* = 0.67) (Figure 8).

### 3.9. Safety

Greater rates of adverse events were observed in the combination therapy groups, compared to the monotherapy groups (RR = 7.91, 95% CI = 1.72–36.43, p=0.008) (Figure S1).

## 4. Discussion

ETV, Lam, or Adv in combination with IFN has become increasingly common in treating CHB patients [26,27]. It has been reported that IFN monotherapy is more efficient than NA monotherapy, leading to HBeAg loss and HBeAg seroconversion. However, the inconvenience of injection limits the use of IFN. Oral NAs can strongly suppress HBV-DNA replication. In addition, combination therapy of IFN and NAs is thought to enhance the therapeutic efficacy [28]. However, it remains inconclusive whether the antiviral efficacy of the combination therapy is significantly better than NA monotherapy. In this study, we used a meta-analysis of clinical data from 17 eligible trials including 2479 patients to compare the antiviral efficacy between the two regimens.

By the end of treatment, our analysis showed the benefits of adding conventional or peg-IFN to NA therapy, as seen by enhanced rates of HBeAg loss (RR = 1.73, *p* < 0.001), undetectable HBV-DNA (RR = 1.87, *p* < 0.001), HBeAg seroconversion (RR = 1.68, *p* < 0.001), and HBsAg loss (RR = 2.51, *p* < 0.001). No significant difference was detected in HBsAg seroconversion, histology improvement, or ALT normalization between combination therapy and NA monotherapy.

Because the combination of NAs with IFN can inhibit more steps of the HBV lifecycle than mainly targeting the reverse transcriptase step by NAs monotherapy, HBV DNA replication can be more efficiently inhibited under the combination therapy. It is expected to see a higher undetectable HBV DNA rate because of the higher viral DNA inhibition efficiency exerted by the combination therapy. Efficient inhibition of HBV DNA replication can reduce intracellular HBV DNA levels, which can lead to reduction and loss of HBeAg, and eventually HBsAg production, resulting in viral clearance in infected cells [29,30]. HBsAg loss would reflect intrahepatic covalently closed circular DNA reduction. Our study showed higher rates of HBsAg loss in the combination therapy group than in the NA monotherapy group, which might be related to the different antiviral mechanisms of these two regimens. The findings of higher HBsAg loss of combination of IFN may be induced by the immunological modulation of the generation of effector T cells in the robust Cytotoxic T lymphocyte (CTL) response [31]. Our analysis demonstrates more efficient inhibition of HBV DNA replication by the combination therapy, which brings about more viral clearance and more frequent reduction of both HBeAg and HBsAg levels secreted into the serum. Thus, differences in antiviral response were detected between the combination therapy and monotherapy groups by this analysis. On the other hand, liver injury as reflected by an elevated ALT level in CHB patients is often associated with a medium range of HBV DNA levels as asymptomatic carriers with high serum HBV DNA levels at the tolerance phase, and inactive carriers with low serum HBV DNA levels after HBeAg seroconversion usually show normal ALT levels. The ALT levels will return to normal once the serum HBV DNA level is reduced to a level less than the middle range. Although we find higher HBsAg loss rate in combination group, we still conclude that no significant difference detected in HBsAg seroconversion, this may because of the comparison time was 48 weeks at the end of treatment. With the longer duration of following up after treatment, HBsAg seroconversion may increase.

HBsAg seroconversion is considered an ideal outcome of anti-HBV therapy, which usually indicates a full viral clearance; however, it occurs infrequently. No significant difference in HBsAg seroconversion was shown between the two groups at the end of treatment, suggesting that it remains a significant challenge to achieve a full viral clearance after one year of treatment by either therapy. Two studies evaluated the histological scores at the end of treatment, and a tendency of improved histology was observed with the combination therapy, although the difference was not significant. We also find there is no significant difference of ALT normalization between two treatment regimens. ALT level at the end of treatment might be influenced by several factors, such as alcoholism, autoimmune and metabolic liver disease, this need to be confirmed by other studies or other meta-analysis.

The studies included in this meta-analysis prescribed different types of IFN and various NAs in the trials. Five studies were based on conventional IFN, and 13 studies were based on pegylated IFN (both conventional and pegylated IFN were mentioned in only one study [11]). Since pegylated IFN was commonly used in later studies, while conventional IFN was often used in earlier studies, it might be difficult to directly compare the efficacies.

The World Health Organization and other HBV guidelines recommend highpotent and low-resistant NAs, or combined with IFN for long-term treatment. The advantages of NA include high rates of HBV suppression and oral administration. The advantages of IFN are the sustained serological response through immune modulation [27,32].

There were several limitations of this study. First, all of the enrolled studies were published in English or Chinese, and no studies in other languages were included. Additionally, we only analyzed the therapeutic efficacy at the end of treatment in this meta-analysis. However, we did compare it both before and after treatment as well as after follow-up. Finally, subgroups between *de novo* and sequential therapeutic groups were not mentioned because of the few available head-to-head studies.

## 5. Conclusions

NA and IFN or Peg-IFN combination therapy results in a better efficacy in terms of HBeAg loss, undetectable HBV-DNA rate, HBeAg seroconversion, and HBsAg loss, compared to NA monotherapy at the end of treatment; however, there was no significant difference in HBsAg seroconversion between the two regimens. In conclusion, combination therapy might be recommended for some patients because of the efficacy and safety at the end of treatment cautiously, but the studies include long-term observation are needed.

## Supplementary Files

Supplementary File 1
