# Supplementary material for: A Better Antiviral Efficacy Found in Nucleos(t)ide Analog (NA) Combinations with Interferon Therapy than NA Monotherapy for HBeAg Positive Chronic Hepatitis B: A Meta-Analysis
Source: Int J Environ Res Public Health. 2015 Aug 21;12(8):10039–55. doi: 10.3390/ijerph120810039 (PMC4555327; doi:10.3390/ijerph120810039)
Supplement: Supplementary File 1 [file ijerph-12-10039-s001.pdf]

Supplementary Information

A Better Antiviral Efficacy Found in Nucleos(t)ide Analog (NA) Combinations with Interferon Therapy than NA Monotherapy for HBeAg Positive Chronic Hepatitis B: A Meta-Analysis

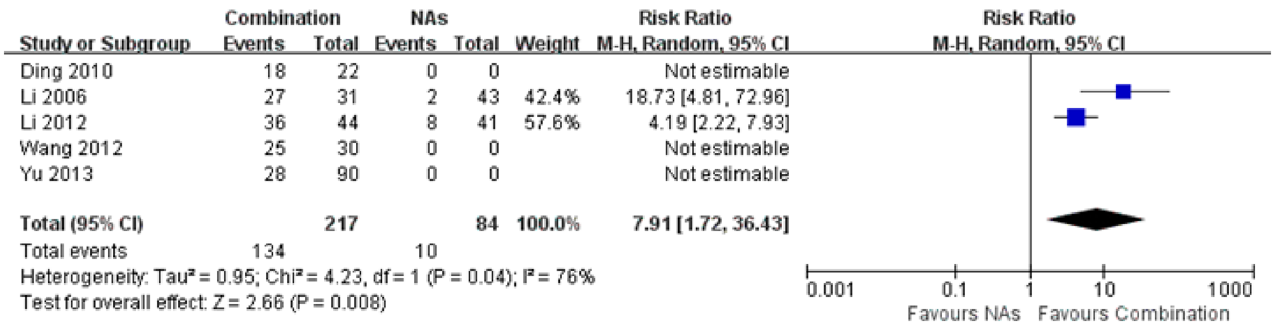

Figure S1. Significant difference in adverse events was identified between the combination and NAs group, and the total risk ratio was 7.91 (95%CI = 1.72–36.43, $p = 0.008$).

© 2015 by the authors; licensee MDPI, Basel, Switzerland. This article is an open access article distributed under the terms and conditions of the Creative Commons Attribution license (http://creativecommons.org/licenses/by/4.0/).
